# Kinetic modeling and parametric imaging with dynamic PET for oncological applications: general considerations, current clinical applications, and future perspectives

**DOI:** 10.1007/s00259-020-04843-6

**Published:** 2020-05-19

**Authors:** Antonia Dimitrakopoulou-Strauss, Leyun Pan, Christos Sachpekidis

**Affiliations:** Clinical Cooperation Unit Nuclear Medicine, German Cancer Research Center, Im Neuenheimer Feld 280, 69120 Heidelberg, Germany

**Keywords:** Dynamic PET, Oncology, Kinetic modeling, Parametric imaging, Feature extraction

## Abstract

Dynamic PET (dPET) studies have been used until now primarily within research purposes. Although it is generally accepted that the information provided by dPET is superior to that of conventional static PET acquisitions acquired usually 60 min post injection of the radiotracer, the duration of dynamic protocols, the limited axial field of view (FOV) of current generation clinical PET systems covering a relatively small axial extent of the human body for a dynamic measurement, and the complexity of data evaluation have hampered its implementation into clinical routine. However, the development of new-generation PET/CT scanners with an extended FOV as well as of more sophisticated evaluation software packages that offer better segmentation algorithms, automatic retrieval of the arterial input function, and automatic calculation of parametric imaging, in combination with dedicated shorter dynamic protocols, will facilitate the wider use of dPET. This is expected to aid in oncological diagnostics and therapy assessment. The aim of this review is to present some general considerations about dPET analysis in oncology by means of kinetic modeling, based on compartmental and noncompartmental approaches, and parametric imaging. Moreover, the current clinical applications and future perspectives of the modality are outlined.

## Introduction

Positron emission tomography (PET) is a tomographic, quantitative, imaging method providing information on biochemical processes in vivo. PET was developed in the late 1970s, and its first applications were brain studies using the radiotracer 2-deoxy-2-(^18^F)fluoro-D-glucose (^18^F-FDG) [1]. In the late 1980s, PET was introduced into oncological studies based mostly on ^18^F-FDG.

The first PET measurements were based on dynamic imaging protocols for the evaluation of tracer pharmacokinetics particularly in the brain. These initial dynamic PET (dPET) acquisitions were confined to a single-bed position, thus limiting their wide clinical application especially in the field of oncology where metastatic disease evaluation calls for whole-body (or at least multibed) PET imaging protocols. Quantitative imaging evaluation was performed by the calculation of time activity curves (TACs), areas under the curve (AUCs), standardized uptake values (SUVs) as well as on more complex kinetic modeling approaches depending on the tracer used. In the case of ^18^F-FDG, the generally accepted method for accurate analysis of the tracer’s kinetics is a compartment model originally proposed by Sokoloff et al., developed to measure 2-deoxy-D-(^14^C)glucose in the rat brain tissue by autoradiography [2]. A few years later, Phelps et al. proposed a modified 3-tissue compartment model for the calculation of the transport rates of ^18^F-FDG, namely *K*_1_ and *k*_2_ which represent the transport rate of ^18^F-FDG into the tissue and reverse, as well as *k*_3_ and *k*_4_ that reflect the phosphorylation and dephosphorylation rate of the tracer. This modified model is actually an extension of the one originally proposed by Sokoloff et al. The difference between them lies in the calculation of *k*_4_ in the model by Phelps et al., which is considered negligible in the Sokoloff model [3]. Since then, several assumptions and modifications have been made to the original mathematical formulations and the model has been successfully applied for the assessment of ^18^F-FDG dPET studies in tumors [4].

Approximately 20 years ago, the conventional PET scanners were replaced by hybrid PET/CT systems, which combine diagnostic information of two modalities. Moreover, the recent development of the novel PET/MRI technology represents a very promising candidate in hybrid imaging, although its role in the clinical setting remains to be determined. The advent of hybrid imaging technology provides superior information due to the combination of structural and functional tomographic imaging modalities. This new era facilitated the performance of whole-body PET/CT scans in a relatively short time—less than 30 min–and with high image quality, leading to the widespread use of the modality in clinical practice. In particular in oncology, PET/CT is considered nowadays the standard imaging technique for diagnosis, staging, and monitoring of several different tumor types.

The vast majority of PET/CT studies are based on acquisition and visual evaluation of static, late, whole-body images usually 60 min post-injection (p.i.) of the radiotracer, occasionally employing semi-quantitative analysis based on calculation of SUV values. SUV represents tissue activity within a region of interest (ROI) corrected for injected activity and body weight, and is the most widely used method for quantification of PET data, since its calculation requires only static imaging, after the tracer is assumed to have reached its equilibrium. SUV calculation is nowadays available in commercial imaging software packages. SUV is, however, dependent on many different factors such as the time interval between injection and scanning as well as different image acquisition settings characteristics (scanner, scatter and attenuation correction, reconstruction algorithm, frame duration), rendering the comparison of SUV values acquired in different centers problematic, when even slight differences in the acquisition procedure are present [5]. Moreover, tracer uptake 60 min p.i. is the result of a dynamic process. One important aspect of PET is the possibility of performing accurate, noninvasive quantitative measurements of tracer concentration in patients, which requires the performance of a dPET study usually with a duration of 60 min, in addition to the regular static PET/CT scan.

Although it is generally accepted that the information provided by dPET is superior to that of conventional static PET acquisitions, several issues have hampered its implementation into clinical routine. Apart from the longer duration of dynamic protocols, some major limitations include the limited axial field of view (FOV) of the current generation clinical PET systems and the fact that the majority of dPET protocols are still confined to a single-bed axial FOV. These issues are beginning to be addressed with the development and introduction of clinical PET systems with gradually longer—more than 1 m—axial FOVs, better electronics, and resolution [6–10]. Moreover, the introduction of clinically feasible dynamic whole-body PET imaging protocols in current generation limited axial FOV PET systems equipped with direct 4D reconstruction schemes and generalized nonlinear graphical analysis methods has rendered graphical analysis (Patlak) whole-body dPET and parametric imaging possible [11–15].

These developments will improve the statistical quality of PET images and allow whole-body scanning in shorter time, and may, subsequently, lead to a potential renaissance of dPET studies with the perspective of even performing whole-body pharmacokinetic studies. dPET scanning can provide reliable assessments of dedicated metabolic steps of metabolic active tracers, such as ^18^F-FDG, as well as newer receptor-binding agents such as radiolabeled prostate-specific membrane antigen (PSMA) or DOTATOC ligands with potential applications in the rapidly evolving field of radiothera(g)nostics. Moreover, the widespread application of dPET would be of great importance in the evaluation of new (radio)pharmaceutical agents.

The aim of this review is to present the general principles of dPET data analysis in oncology by means of kinetic modeling, based on compartmental and noncompartmental approaches, as well as by parametric imaging. Moreover, the current clinical applications and future perspectives of dPET will be outlined.

### dPET studies: general considerations

#### Volume of interest–based analysis

##### Workflow, protocol

**Fig. 1:**
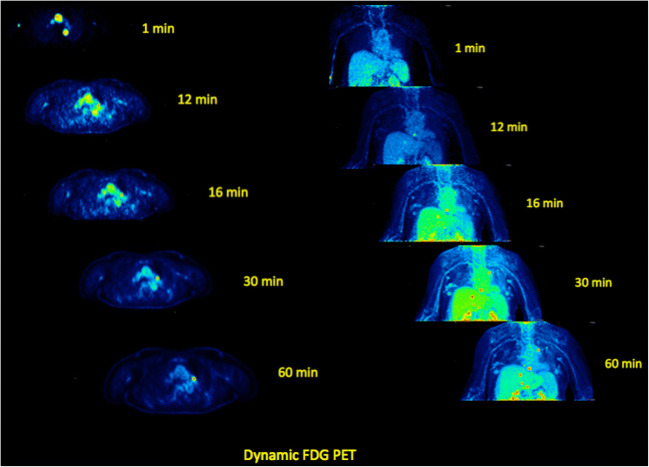
Transversal (left) and coronal (right) images of a dPET series of the thorax following i.v. ^18^F-FDG injection at 1, 12, 16, 30, and 60 min p.i. in a patient with metastases (mediastinal, lung, and liver) from melanoma. Visualization of the vessels in the early images and gradually increasing ^18^F-FDG uptake in the metastases in the following frames

dPET studies require a dynamic acquisition for a certain time depending on the pharmaceutical and the radionuclide used. The target area for dPET should be defined carefully, usually including the anatomical region with the known or suspicious tumor lesions. The advent of the new-generation PET scanners with extended FOV will simplify the choice of a target area by allowing dynamic acquisition of almost the whole body. For ^18^F-FDG, the most common used PET tracer in oncology, 60 min of dynamic acquisition are required. Similarly, 60-min dynamic acquisitions are also applied for receptor-binding tracers, such as DOTATOC and PSMA radioligands [16, 17]. For transport tracers, like ^11^C-labeled amino acids (e.g., C-11-methionine or C-11-choline)) a shorter acquisition protocol, e.g., for 20–30 min, is usually applied. The dynamic imaging is acquired in a list mode, and then, frames are defined by the users (Fig. 1). The frame duration should be short for the first frames and increase during the progress of the dPET acquisition (e.g., 10 s, followed by 30 s, 60 s, 120 s, and 300 s).

##### Definition of tumor VOIs

The evaluation of the reconstructed PET images is based on visual analysis of PET and fused PET/CT or PET/MRI images. Tumor volume of interest (VOI) is then placed over areas with increased tracer uptake as compared with the surrounding tissue, after correlation with the CT/MRI images and using dedicated algorithms, usually an isocontour. VOIs drawn over reference tissue without pathologically increased tracer uptake are also recommended for comparison.

##### Input function: general considerations and problems

The accurate assessment of the arterial input function is a topic broadly investigated in the literature. Manual blood sampling at different time points p.i. or continuous automatic blood sampling during the whole dynamic acquisition have been used in limited number of studies [18]. Phelps et al. first reported on a good agreement between arterial blood sampling and arterialized venous sampling by using hand heating to 44°C [3]. Although, arterial blood sampling is considered the gold standard for input function measurements, it is an invasive procedure and cannot be recommended for routine clinical purposes. Furthermore, delay in terms of different appearance time of the radioactivity after a bolus injection and dispersion affects the time activity curve used for input. In particular, when using blood sampling, the measured blood curve is smeared due to inhomogeneous velocity in the vessels and the catheter and due to sticking of radiotracer in the catheter tubes used. Therefore, dispersion correction is recommended in particular for tracers with very fast kinetics, like perfusion studies [19, 20].

Double supply of organs like the liver causes special problems in the estimation of the input function. In particular, the liver receives blood supply from both the portal vein and the hepatic artery with the TAC of the portal vein being delayed and dispersed as compared with the hepatic artery. Keiding reported on methods of estimating the dual-input TAC without portal vein measurements in particular for measurements of the regional hepatic blood perfusion [21].

Noninvasive devices have been introduced for the determination of the arterial input function. Recently, Turgeon et al. introduced a detector of scintillating fiber coupled to transmission fiber-optic cables, which are connected to photomultiplier tubes as a device that can be wrapped around the wrist of the patient studied with PET [22]. The results are promising and suggest that scintillating fibers may be used for the noninvasive measurement of the arterial input function.

**Fig. 2:**
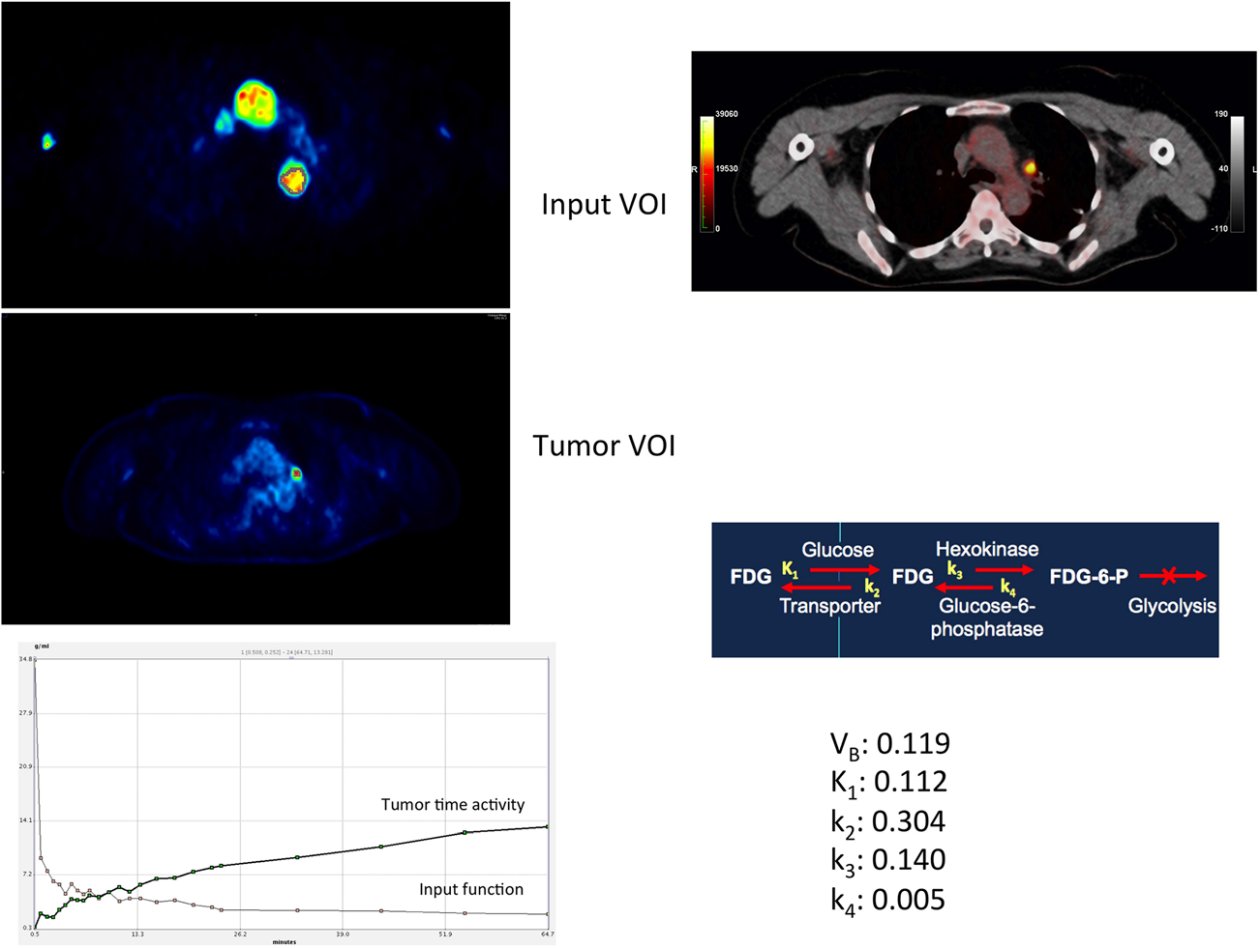
Patient of Fig. 1. Fused ^18^F-FDG PET/CT late image (upper right) approximately 80 min p.i. demonstrating an ^18^F-FDG avid mediastinal lymph node metastasis. Input and tumor VOIs in the descending aorta and the mediastinal lesion for evaluation of the kinetic data of the tracer (left upper and middle row, respectively). Time activity curves of both VOIs and results of kinetic analysis based on a 3-tissue compartment model (lower row)

However, the most attractive, easy, und user-friendly approach for input function assessment, which also possesses a good accuracy, is its image-derived calculation during the PET scan [23]. Input VOIs can be acquired using the reconstructed images of the first frame(s) drawn over a large arterial vessel like the descending aorta (Fig. 2). An input VOI should include the hottest pixels of the vessel in several sequential slices. Visual inspection should then be used for a first qualitative assessment of the respective TACs: an input VOI should demonstrate a clear activity peak and not be very noisy. In case of much noise, for example due to the use of a smaller vessel, the curve data should be fitted by using a sum of up to three decaying exponentials to reduce noise [24, 25]. For smaller vessels (diameter < 8 mm), like the femoral artery, a partial volume correction may be done using the CT data and on the basis of phantom measurements of the recovery function. In recent years, novel approaches for calculation of image-derived arterial input function using integrated PET/CT and PET/MRI images have been developed with very promising results [26, 27].

Another approach is the use of a standardized, population-based input function, which is a normalized average of measured arterial blood samples from several subjects [28, 29]. Furthermore, hybrid statistical approaches have been published for the noninvasive assessment of the input function. O’Sullivan et al. proposed a penalty formulation in which the information derived from priori studies is combined in a Bayesian manner with information contained in the sampled image data and recommend this method for clinical use [30].

##### Lumped constant

The difference between ^18^F-FDG and glucose in terms of transport, phosphorylation, and distribution volume is taken into consideration by using a correction term, the so-called lumped constant (LC_FDG_). The accuracy of the metabolic rate of ^18^F-FDG depends on the knowledge of the exact value of LC. Sokoloff et al. measured the LC for ^14^C-deoxyglucose in the rat brain and reported a value of 0.46 [1]. Reivich et al. found a value of 0.52 in human brain [31]. Hasselbalch et al. reported on a mean value of 0.81 ± 00.15 in normal healthy volunteers [32]. However, for simplification reasons, LC is considered to be equal to one.

#### Compartment modeling

**Fig. 3:**
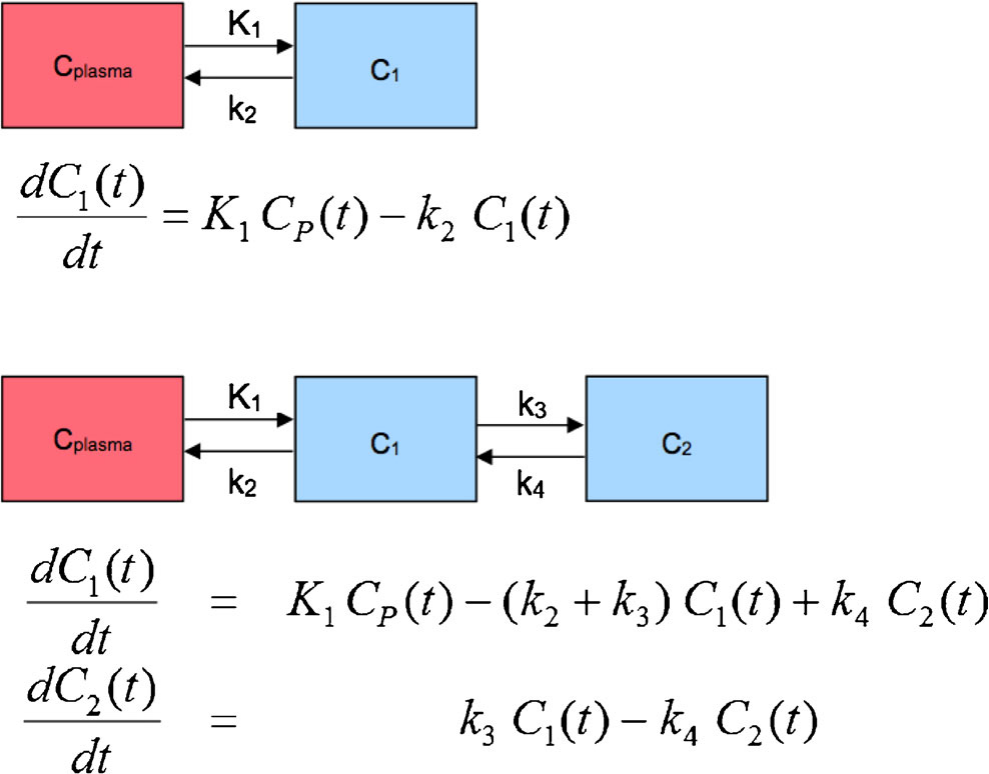
Schematic presentation of the 2- and 3-compartment model with one input function. *C*_plasma_ is the tracer concentration in arterial blood. In the 2-tissue compartment, all tracer is transported in compartment *C*_1_. For the 3-tissue compartment, two tracer forms in tissue are considered as *C*_1_ and *C*_2_. Compartment *C*_1_ represents the free and nondisplaceable part of the tracer into the tissue, and compartment *C*_2_ represents the specific bound part of the tracer (in case of ^18^F-FDG the phosphorylated tracer)

The idea of compartment modeling of PET data has its roots in pharmacology and biochemistry. By providing information on the transport or other metabolic steps of the applied radiopharmaceutical, compartment modeling aims to estimate biologically relevant parameters. However, in vivo dynamic imaging is more complex than in vitro studies partly due to the fact that the uptake of a radiopharmaceutical depends on the vessel density of a tissue. The most commonly used compartment models in PET are the 2-tissue and 3-tissue compartment models (Fig. 3). A simplification of these models consists of the summation of the interstitial and the cellular space. A 2-tissue compartment model is suitable for radiopharmaceuticals, which are purely transport markers, like labeled water or amino acids that do not undergo further metabolic steps. A 3-tissue compartment model is appropriate for radiopharmaceuticals, which are transported and then undergo one metabolic step, like ^18^F-FDG. This model involves the plasma compartment C_p_, the exchanging compartment C_1_ in which the tracer is considered free and nonspecifically bound tracer in tissue (nondisplaceable compartment), and the compartment C_2_ involving the specifically bound tracer. The arterial plasma C_p_ exchanges with the first tissue compartment C_1_, which in turn exchanges with the second tissue compartment C_2_. The application of the 3-tissue compartment model leads to the extraction of the kinetic parameters *K*_1_ and *k*_2_, which are the uptake and clearance rate constants (i.e., between *C*_*p*_ and *C*_*1*_), as well as of the parameters *k*_3_ and *k*_4_, which describe the exchange between the tissue compartments *C*_1_ and *C*_2_ (Fig. 3) (http://doc.pmod.com/pkin/pkin.html). The unit for these rate constants is 1/min.

In the case of ^18^F-FDG, it is known that the tracer is transported by different glucose transporters, phosphorylated by different hexokinases and then trapped intracellularly without undergoing further metabolic steps [33, 34]. According to the 3-tissue compartment model, four transport rates describe the exchange of the radiopharmaceutical between the blood and the tissue compartments. Appropriate algorithms, like Marquardt-Levenberg, allow an estimation of the fractional blood volume, also known as vessel density (*V*_*B*_), as well as the above-described transport rates of ^18^F-FDG *K*_1_, *k*_2_, *k*_3_, and *k*_4_: *K*_1_ is related to the influx of the tracer from the blood compartment to the tissue compartment, *k*_2_ is related to the efflux, *k*_3_ reflects the phosphorylation rate, and *k*_4_ reflects the dephosphorylation rate. *V*_*B*_ and *K*_1_ are related and are typically higher than the phosphorylation rate *k*_3_. One should keep in mind that the rates assessed by the compartment modeling are a compromise between a pure mathematical solution and biological and practical limitations of the method. It is recommended to use an upper limit of 1 for each transport rate, while *V*_*B*_ values should exceed 0, even if from a mathematical point of view different values may be occasionally calculated. This model is different than the one proposed by Sokoloff et al., which does not take into consideration *k*_4_ and *V*_*B*_. The lack of *k*_4_ and *V*_*B*_ leads to different *K*_1_ and *k*_3_ values, since *K*_1_ is dependent on *V*_*B*_, and *k*_3_ dependent on *k*_4_. Moreover, the dephosphorylation rate (*k*_4_) of ^18^F-FDG may be low, but is not negligible. Details about the implementation of different compartment models and the software requirements for clinical use are described by Burger and Buck [35] (https://www.pmod.com/web/?portfolio=11-modeling-pkin). Of note is that this model may be confounded in kidney evaluations by urinary excretion.

A limitation of compartment modeling is that the assessment of the transport rates is operator-dependent and should be performed only by experienced users. The reason is that these models use an iterative fitting (IF) to calculate the least squares between measured and model data, which may lead to overfitting problems and lack of reproducibility. Noise in the TACs and, in particular, inappropriate input TACs have an impact on the assessed rates. A solution to overcome these problems has been published by a group, based on machine learning approaches and oncological reference databases with a training set of modeling data. In specific, we have introduced a machine learning (ML)-based kinetic modeling (KM) method, that utilizes a historical reference database to build a kinetic model directly dealing with noisy data but not trying to smooth image noise. Based on the plethora of data in the reference database, this approach can automatically adjust the models using a multithread grid parameter searching technique. Moreover, in an attempt to combine the advantages of ML and IF modeling methods, a candidate competition concept has been developed, which can find a balance between fitting to history data and to unseen target curve. The ML-based method provides a robust and reproducible solution that is user-independent for VOI-based and pixelwise quantitative analysis of PET data [36].

**Fig. 4:**
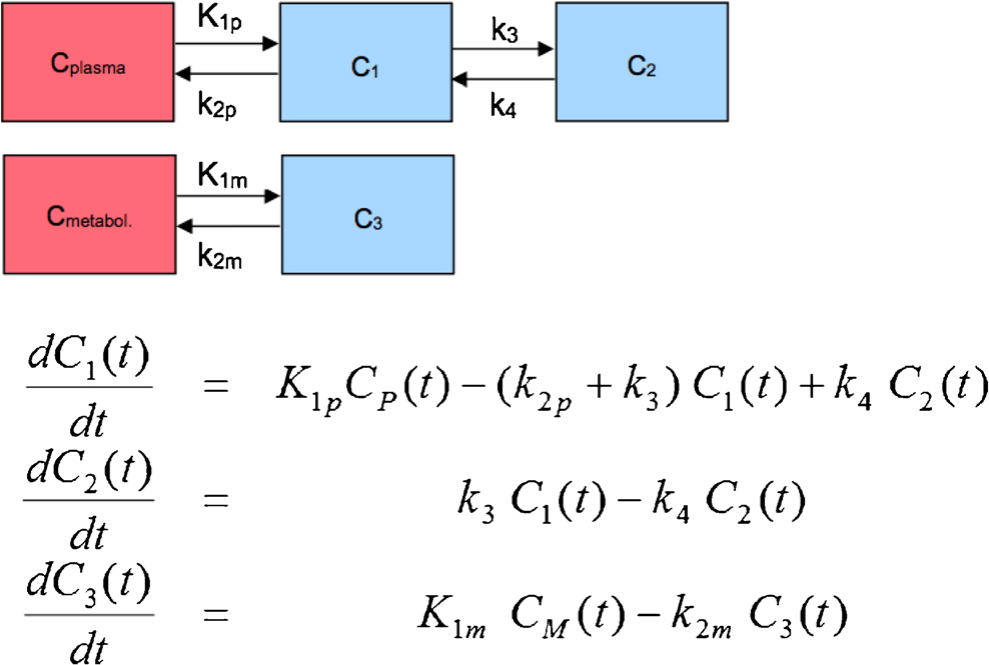
Schematic presentation of a 3-compartment model with a double input function. If a labeled metabolite of the tracer enters tissue, the additional signal has to be accounted for in the model. This model includes a second input curve *C*_*M*_(*t*) of a metabolite entering tissue and undergoing nonspecific binding. *C*_1_ represents the nondisplaceable compartment of the authentic ligand, *C*_2_ the specific binding of interest, and *C*_3_ metabolized ligand in tissue. *C*_*P*_(*t*) and *C*_*M*_(*t*) are the input curves of authentic ligand and metabolite, respectively. *C*_*p*_ *C*_plasma_, *C*_*M*_ *C*_metabolite_

Other, more complicated models with two input functions and five compartments have also been proposed for metabolically active tracers, like ^11^C-thymidine, ^11^C-acetate, or ^18^F-fluorodeoxythymidine (FLT) (Fig. 4) [37]. These tracers produce labeled circulating metabolites during the dynamic data acquisition due to biochemical breakdown or conjugation shortly after injection. The measurement of these circulating metabolites and the knowledge of their biological behavior in terms of the metabolic processes in which they may be involved is a challenge. Therefore, model input functions of not only the injected radiotracer but also its labeled metabolites should be used for more accurate quantitative assessment. Such a model example is provided for ^18^F-FLT and its circulating metabolite ^18^F-FLT-glucuronide by Muzi et al. [38]. However, these approaches are very complicated and, thus, not recommended for clinical use. In an attempt to address this issue, more simplified models have been proposed for the characterization of these agents. For example, Shields et al. assessed a 3-tissue compartment model for ^18^F-FLT with an image-derived input function of the descending aorta and metabolite correction measured in a single sample obtained at 60 min p.i. They reported that at 60 min p.i., 74% of the blood activity was unmetabolized. The authors found a good correlation (*r*^2^ = 0.82) between the image-derived input function and the venous blood sampling, while the metabolic rate of the tracer correlated strongly with average SUV (*r*^2^ = 0.85) [39]. Other modifications have been proposed for a mathematical metabolite correction of receptor data by Burger and Buck using a series of ^11^C-iomazenil patient PET data [40]. Reference tissue compartmental methods have been proposed for the estimation of the binding potential from reversible ligand-receptor PET studies. In this case, instead of the use of an input VOI in a vessel, a reference region with no or very low specific uptake is used. Details about reference tissue compartmental modeling have been reported in the literature [41, 42]. An overview of the different algorithms, which are in use for compartment modeling is provided elsewhere [43].

#### Patlak-Gjedde plot

The Patlak plot, also known as Patlak-Gjedde plot, is a graphical analysis technique based on a compartment model that uses linear regression to analyze the pharmacokinetics of a tracer. Prerequisite for the use of a Patlak plot is an irreversible trapping compartment of the tracer, as is assumed to be the case for ^18^F-FDG [44]. The model is based on a blood/plasma compartment, a reversible and a nonreversible compartment, and can demonstrate whether the major metabolic step fits to a unidirectional transfer of the tracer, allowing the graphical calculation of an influx constant (*K*_*i*_). Importantly, since most biological reactions do not occur in a completely irreversible manner, Patlak and Blasberg had allready proposed in 1985 also a generalized version of this model, which could tolerate mild degrees of reversibility (*k*_4_ > 0) to avoid underestimation of the net uptake rate constant *K*_*i*_ as previously reported for ^18^F-FDG in certain regions, such as the normal liver, and tumor types, such as hepatocellular carcinoma [44]. This generalized version of the Patlak model was recently successfully applied for whole-body dPET and parametric imaging [45, 46].

The Patlak plot is calculated according to the formula:

$$ \frac{C_T(t)}{C_P(t)}=K\frac{\underset{0}{\overset{t}{\int }}{C}_P\left(\tau \right) d\tau}{C_P(t)}+V $$

with *C*_*P*_(*t*) representing the input curve, *C*_*T*_(*t*) the measured tissue TAC, *K* the slope, and *V* the intercept.

The interpretation of *K* and *V* is based on the underlying compartment model. Particular in ^18^F-FDG, *K* equals *K*_1_ × *k*_3_ / (*k*_2_ + *k*_3_) and represents the metabolic flux of the tracer, while *V* equals *V*_0_ + *V*_*B*_, where *V*_0_ represents the distribution volume of the reversible compartment *C*_1_ and *V*_*B*_ the fractional blood volume.

Moreover, the analysis of ^18^F-FDG data requires the lumped constant (LC) and the plasma glucose level (PG) of the patient. The metabolic rate of glucose MRGlu is subsequently obtained from the regression slope by

$$ \mathrm{MRGlu}={K}_i\frac{\mathrm{PG}}{\mathrm{LC}} $$

#### Noncompartmental models

##### Fractal dimension

Besides compartment analysis, a noncompartment model based on the fractal dimension (FD) can be applied. Fractal geometry is used to quantify structures that are poorly represented by the Euclidean geometry, for example for quantification of lesions with high structural complexity and irregular borders, and might therefore be helpful as an additional classification parameter [47].

FD is a parameter of heterogeneity and can also be used for temporal series, such as the time-activity data in each individual voxel of a VOI. The values of FD vary from 0 to 2 showing the deterministic or chaotic distribution of the tracer activity, with higher values reflecting more heterogeneous tracer distribution. Our group has evaluated TAC data derived from ^18^F-FDG PET studies in 159 patients with 200 malignant lesions of different tumor entities as well as in 57 patients with 57 benign lesions for comparison. We used a box counting procedure as well as a subdivision of 7 × 7 and a maximal SUV of 20 for the calculation of FD, and found that FD demonstrated an accuracy of 77% for all patients, 68% for the untreated, and 83% for the treated group [48]. A FD cutoff value of 1.13 could reliably discriminate between malignant and benign lesions.

Fractal analysis has been furthermore used to quantify static whole-body images of patients with metastatic melanoma for immunotherapy monitoring after ipilimumab monotherapy [49]. In particular, the spreading of tumor cells was modeled via Monte Carlo simulations to address the evolution of the metastatic process and to predict the spatial distribution of metastatic lesions. Interestingly, FD was shown to decrease consistently with disease progression. Notably, problems existed in case of inflammatory lesions, like immune-related adverse events (e.g., colitis, thyroiditis), suggesting that areas with unspecific, nontumor–related uptake should be excluded for FD calculations. Overall however, the method is robust and operator independent and may be used as an additional tool for a multiparametric, PET-based, oncological assessment.

#### Parametric imaging—pixelwise modeling

Parametric imaging is a method of feature extraction allowing the visualization of an isolated parameter of a tracer’s kinetics based on dedicated mathematical models and a voxelwise calculation, instead of a VOI-based analysis. Parameters, which can be visualized, are the perfusion-related part of a tracer (transport), further metabolic steps—such as phosphorylation in the case of ^18^F-FDG—as well as the global influx. In case of receptor-specific tracers, parametric images of the receptor-binding or the internalization of the agent can be calculated. The advantage of parametric imaging as compared with the VOI-based image analysis is the calculation of images instead only of numbers. The most common approach is the usage of the reconstructed PET images and post-processing of the data based on different algorithms. The first results of parametric imaging were published in 1992 by Messa et al. in patients with liver metastases [50].

##### Simplified parametric analysis of dPET data

**Fig. 5:**
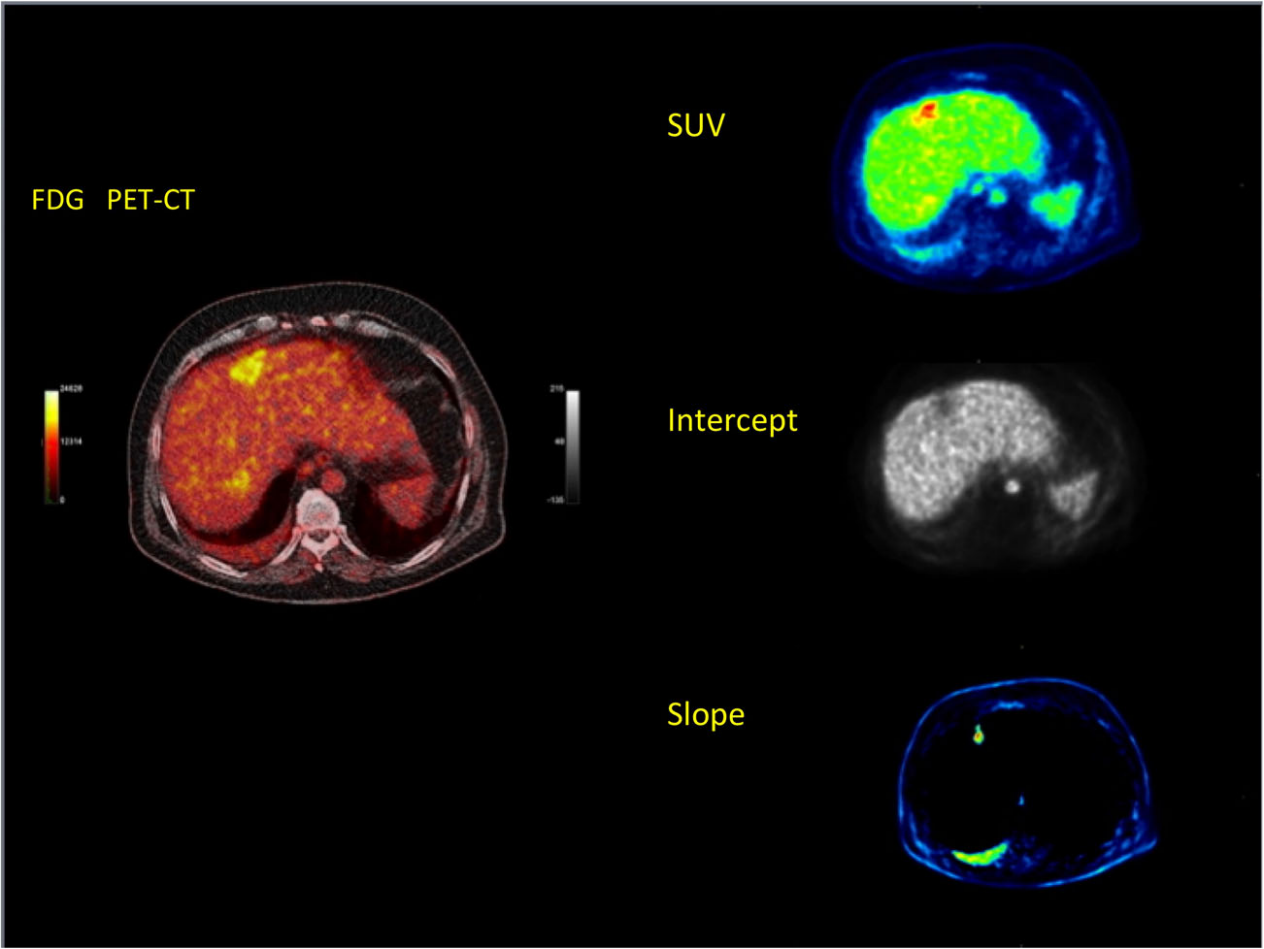
Patient with a liver metastasis of rectal cancer following FOLFOX chemotherapy. Fused transversal ^18^F-FDG PET/CT image (left) demonstrating enhanced uptake at the site of the metastasis 60 min p.i. Transversal SUV image 50–60 min p.i. demonstrates an enhanced uptake (right upper row), while parametric image of the intercept (middle row) shows a decrease in the perfusion-related ^18^F-FDG uptake, and parametric image of the slope (lower row) demonstrates an enhanced phosphorylation-related ^18^F-FDG uptake

Parametric images can be calculated by fitting a linear regression function to the time-activity data on a pixel basis. Images of the slope and the intercept can then be calculated using dedicated software packages, such as the PMod software (PMOD Technologies Ltd., Zuerich, Switzerland) [51]. In particular, parametric images of the slope reflect primarily the transport/phosphorylated part of ^18^F-FDG and may be used for the delineation of suspicious tumor lesions due to the better contrast as compared with the SUV images (parametric images calculated from the original DICOM images by dividing them by injected dose normalized to body weight) or for the VOI placement in order to assess the trapped (phosphorylated) part of ^18^F-FDG in a lesion. Respectively, parametric images of the intercept reflect the transport/perfusion-related part of ^18^F-FDG, which is an indirect parameter of the perfusion of a lesion (Fig. 5). Some tumors, such as the giant cell tumors, are very clearly delineated in the intercept images of ^18^F-FDG. It was shown, that the enhanced perfusion related part of the ^18^F-FDG in these tumors correlated to enhanced expression of genes related to angiogenesis, like the vascular endothelial growth factor A [52]. Futhermore, intercept images allow the visualization of vessels and can be used for the placement of input VOIs. Another advantage of this simplified parametric analysis of dPET data is that no input function is needed. Details of this method have been described elsewhere [53].

##### Patlak-based analysis

Similar to compartmental modeling and in contrary to the simplified parametric analysis of dPET data, an input function is needed for the calculation of influx and intercept images according to Patlak analysis. Furthermore, the time frame used for the calculations should be defined by the operator. In general terms, it is recommended that the linear part of the TAC of a radiotracer should be used for Patlak analysis, when all reversible compartments are in equilibrium with plasma. In the case of ^18^F-FDG this is approximately fulfilled during the last 30 min of the 60-min dPET acquisition [44]. However, several investigators have used the dynamic data from the whole dPET acquisition for the calculation of parametric Patlak images, resulting in differences depending on the time frame used for the calculation. A dependence of the Patlak slope and intercept parameters and the post-injection time window of the dPET data from which the Patlak parameters were estimated, has been demonstrated. This time dependence of the supposedly time rate constant parameters of the Patlak model suggest that the conventional linear Patlak model may not always be the appropriate model for accurately describing the underlying tracer kinetics, for example due to the presence of nonnegligible uptake reversibility by the tracer under study [45, 54].

**Fig. 6:**
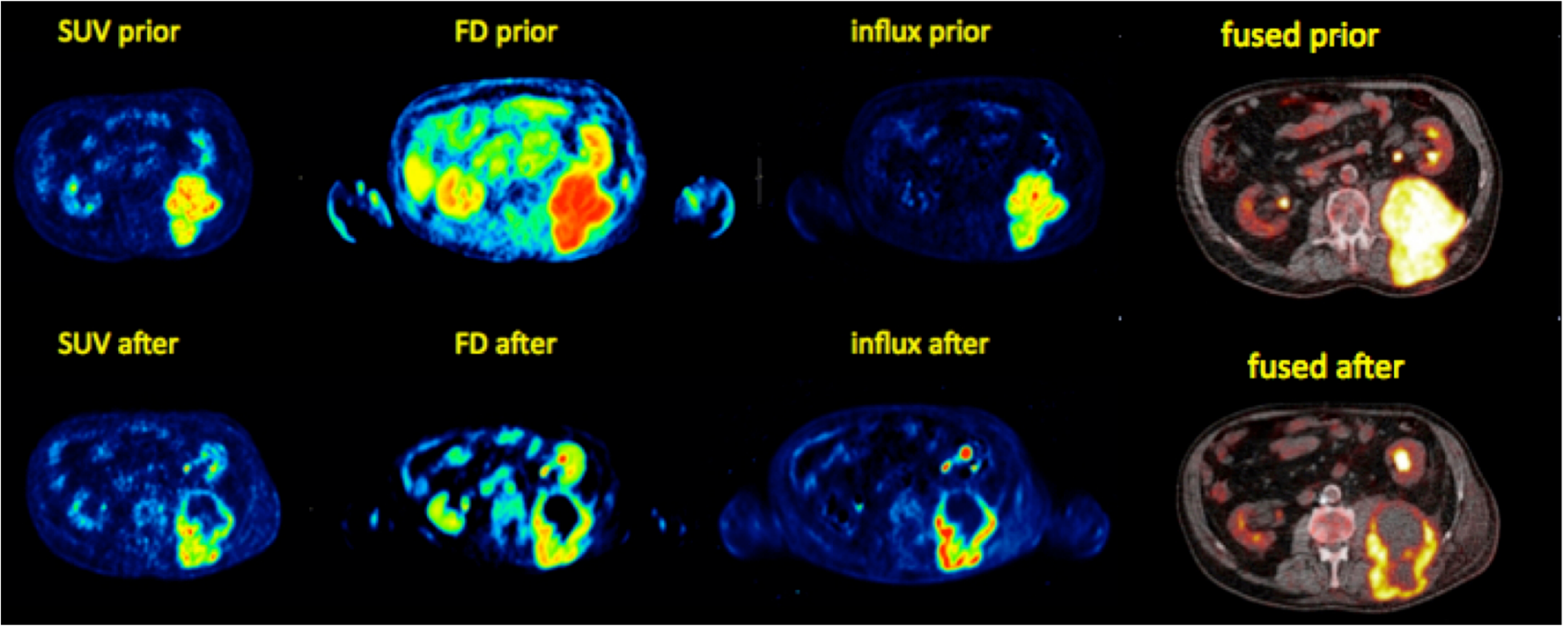
Patient with an advanced high-grade retroperitoneal sarcoma infiltrating the left dorsal muscles. Tranversal ^18^F-FDG PET images prior (upper row) and after the end of neoadjuvant pazopanib therapy (lower row) on the left part of the figure. The 55–60-min SUV images demonstrate a decrease of uptake and a central tumor necrosis as response to therapy (left). Comparable ^18^F-FDG response pattern in the parametric images of the fractal dimension and the influx according to Patlak (middle). “Conventional,” tranversal-fused PET/CT images on the right part

The intercept and influx images according to Patlak are related and very similar to the intercept and slope images based on the simplified parametric analysis model (Fig. 6).

Besides the standard linear Patlak graphical analysis, which does not take into account uptake reversibility, e.g., dephosphorylation in the case of ^18^F-FDG, a nonlinear generalized Patlak method has been introduced by Patlak and Blasberg [44]. Moreover, Karakatsanis et al. proposed different Patlak-based parametric imaging methods for multibed dPET imaging. They compared the linear with the nonlinear graphical analysis and introduced a hybrid method consisting of a combination of linear and nonlinear Patlak analysis; this was achieved by applying nonlinear Patlak analysis selectively only to TACs from voxels exhibiting high Patlak correlation coefficients and linear Patlak to all other voxels. The authors recommend the use of nonlinear Patlak for highly quantitative imaging tasks, while for lesion detectability the hybrid technique is superior. Overall, all methods resulted in higher contrast-to-noise ratios as compared with SUV images [45].

In 2004, Zhu et al. developed a 4D linear Patlak algorithm for direct reconstruction from list-mode data across multiple bed positions based on a dual time point dynamic protocol instead of a full multiframe dynamic scan [55]. Recently, whole-body direct 4D parametric PET images employing nested generalized Patlak expectation-maximization reconstruction algorithms have been introduced and the first results demonstrate less noise in K_i_ than the conventional post-processing Patlak images [46]. The authors of this study propose a 7-frame protocol, consisting of an initial early dynamic scan (6 min) at the cardiac bed position immediately after the tracer injection for the calculation of the image-derived input function, followed by 6 whole-body dynamic scans over seven bed positions (40 min). However, the procedure needs a high computer capacity and a long reconstruction time. Some recently published studies have highlighted the clinical feasibility and impact of these approaches [14, 56].

##### Two- and three-tissue compartment model

It is possible to calculate parametric images of the transport rates *K*_1_, *k*_2_, *k*_3_, and *k*_4_ as well as of the distribution volume *V*_*B*_ based on a voxel-based application of the 3-tissue compartment described previously in this article. However, the robustness of the method is limited and needs further development. The advantage of the visualization of the transport rates and the distribution volume is the direct visual comparison of the rates in particular in follow-up studies, and theoretically the direct VOI-based calculation of the rates using the parametric images of each rate.

##### Fractal dimension

Parametric images of FD can also be calculated [48]. As mentioned above, fractal geometry can be used to quantify lesions with high structural complexity and irregular borders, and might therefore be helpful as an additional parameter for the assessment of tracer heterogeneity in PET images. The example of Fig. 6 demonstrates a marked FD enhancement within a sarcoma of the left back muscles on the parametric image of FD, which is indicative for an enhanced heterogeneity in the tumor area (Fig. 6).

##### Principal component analysis

Principal component analysis (PCA) is another noncompartment model, which describes the variance-covariance structure of a set of variables through some linear combinations of them. PCA has the main objectives of data reduction and interpretation and can be used for parametric imaging. This method visualizes regions with different kinetics in a dynamic sequence by explaining the variance-covariance structure of the data set, leading to optimization of the signals by simultaneously considering the complete set of images in the dynamic sequence. Being independent of any kinetic model, PCA does not require the manual selection of VOIs and does not include any model-based restrictions. In general, only the first two to three components are useful for image interpretation: the first component is related to the perfusion in the case of ^18^F-FDG, whereas the second one is related to enhanced viability [57]. Methods for reduction of the background noise have been proposed to increase the image quality [58].

##### Similarity mapping

Similarity mapping (SM) is a method of segmenting images into regions according to their temporal rather than spatial properties. Based on this approach, the similarity between the TAC of each pixel and the TAC of a reference ROI is calculated and displayed as an image. SM images provide spatially differentiated quantitative information describing the physiological behavior of the image structures, which sometimes may not be easily extracted from the visual inspection of dPET image sequences. Our group has reported on the application of this method in 20 patients with different malignancies who underwent ^18^F-FDG dPET [59]. In that study, SM supported the visual interpretation of PET data: based on the squared-sum normalized correlation coefficient, SM could identify structures with similar temporal properties to the tumor, enhancing the detection of metastases that were not easily depicted in the SUV images due to poor image quality or lesions’ characteristics (size, location, etc.).

### Current clinical applications

^18^F-FDG PET/CT imaging has a great impact on the diagnostics and management of oncological patients and has gained tremendous use worldwide [60, 61]. As mentioned above, most of the oncological studies are performed with static, whole-body PET/CT acquisitions, which assessment is mostly based on visual analysis and semi-quantitative evaluations by means of SUV calculations. In contrast, dPET imaging has been traditionally used for research purposes and is, thus, mainly performed in dedicated centers. A PubMed literature search with the keywords: compartment AND patients AND tumor AND PET revealed 204 papers (until October 2019). After exclusion of case reports, review articles and articles published in other languages than English 133 articles remained. The majority of these articles (*n* = 42) were based on dPET studies with ^18^F-FDG and used 3-tissue compartment modeling, Patlak analysis, and fractal analysis. An overview of the radiotracers used, the tumors studies, the model used for kinetic analysis, and the goal of these studies is presented in Table 1.

**Table 1:** Summary of the published dynamic PET studies in oncology applying kinetic modeling and/or parametric imaging

Tracer	No. of papers	No. of patients	Tumor type	Model	Goal
^11^C-acetate	3	6–22	PC, HCC	3-Tissue, Patlak	Kinetics, model comparison
^11^C-choline	2	19–194	PC	2-Tissue, 3-tissue, Patlak	Kinetics, comparison tumor to hyperplasia
^11^C-erlotinib, ^15^O-H_2_O	1	10	NSCLC	3-Tissue, 2-tissue	Kinetics, comparison of tracers, therapy response
^15^O-H_2_O	5	6–23	Different tumors	2-Tissue, modified 2-tissue, parametric imaging	Kinetics, comparison of tracers, therapy response
^15^O-H_2_O and ^18^F-FDG	3	16–35	Different tumors	2-Tissue, 3-tissue, Patlak	Kinetics, comparison of tracers, therapy response
^11^C-methylglucose and ^18^F-FDG	2	7–40	Brain	3-Tissue	Kinetics, tracer comparison
^11^C-methionine	1	14	Brain	3-Tissue	Kinetics
^11^C-thymidine	1	20	Brain	5-Tissue	Kinetics, comparison with clinical and pathological data
^11^C-tyrosine	1	11	Brain	5-Tissue	Kinetics
^18^F-choline	2	10–14	PC, different tumors	2-Tissue, 3-tissue, modified nonlinear compartment	Kinetics, comparison with multiparametric MRI
^18^F-DCFPyL	1	3	PC	3-Tissue	Kinetics
^18^F-FDOPA	2	9–37	Pheochromocytoma, paraganglioma, brain	2-Tissue, 3-tissue, Patlak	Kinetics, model comparison, correlation to grading, differential diagnosis
^18^F-FAZA	3	9–20	Pancreas, NSCLC	3-Tissue, Patlak, spectral analysis, parametric images	Kinetics, model comparison
^18^F-FDG	41	up to 117 with full dynamic	Different tumors	3-Tissue, Patlak, fractal, PCA, parametric imaging	Kinetics, model comparison, diagnosis, therapy response, correlation to grading, correlation to gene expression data
^18^F-FDG	1	217 with a short protocol	Breast	First-pass Mullani model, texture analysis	Perfusion based part of FDG, correlation to heterogeneity, receptor expression
^18^F-FLT	12	8–39	Breast, colorectal, brain, head and neck, lung, other	3-Tissue	Kinetics, model comparison, therapy response, comparison to proliferation
^18^F-NaF	1	80	MM	3-Tissue, fractal	Kinetics, diagnosis
^18^F-FDG and ^18^F-FLT	2	8–15	MM, brain	3-Tissue, fractal	Kinetics, tracer comparison, diagnosis,
^18^F-FDG and ^18^F-NaF	2	34–60	MM	3-Tissue, fractal	Kinetics, tracer comparison, therapy response
^18^F-FDG and ^15^O-H_2_O and ^18^F-EF5	1	22	Head and neck	2-Tissue	Kinetics, correlation of hypoxia to clinical outcome
^18^F-DOPA and ^18^F-FDG and ^15^O-H_2_O	1	11	Melanoma	2-Tissue, 3-tissue	Kinetics, tracer comparison, diagnosis
^18^F-DOPA and ^18^F-FLT	1	21	Brain	3-Tissue, factor analysis	Kinetics, model comparison, therapy response evaluation
^18^F-DOPA and ^18^F-FET	1	16	Brain	3-Tissue	Kinetics, tracer comparison, comparison with grading
^18^F-FET	4	7–16	Brain	2-Tissue, 3-tissue, Patlak, spectral analysis	Kinetics, model comparison, comparison with clinical outcome
^18^F-fluciclovine and ^11^C-methionine	2	6–27	Brain	2-Tissue, 3-tissue	Kinetics, tracer comparison
^18^F-fluorocholine	1	8	PC	2-Tissue, 3-tissue	Kinetics, model comparison
^18^F-FDHT and ^15^O-H_2_O	1	71	PC	Patlak	Kinetics, tracer comparison
^18^F-fluoroglutamine	1	41	Different tumors	3-Tissue	Kinetics
^18^F-FMAU	1	10	Brain, PC	3-Tissue	Kinetics, model comparison
^18^F-FMISO	9	6–120	NSCLC, head and neck	2-Tissue, 3-tissue, 4-tissue	Kinetics, model comparison, therapy response, comparison to CT and MRI
^18^F-FMISO and ^15^O-H_2_O	1	11	Brain	2-Tissue, 3-tissue	Kinetics, model comparison, tracer comparison
^18^F-FMISO and ^18^F-FDG	1	13	NSCLC	3-Tissue, fractal	Kinetics, model comparison, tracer comparison
^18^F-galacto-RGD	1	19	Different tumors	3-Tissue	Kinetics
^18^F-HX4	1	8	NSCLC	3-Tissue	Kinetics
^68^Ga-bombesin	1	7	Brain	3-Tissue, fractal	Kinetics, comparison with gene data
^68^Ga-bombesin & ^18^F-FDG	2	15–17	GIST, brain	3-Tissue, fractal	Kinetics, tracer comparison, diagnosis
^68^Ga-DOTATOC	2	21–22	NET, meningioma	3-Tissue, fractal	Kinetics, tracer comparison, diagnosis
^68^Ga-DOTATOC and ^18^F-FDG	2	9–15	NET, NSCLC	3-Tissue, fractal	Kinetics, tracer comparison, diagnosis
^68^Ga-DOTATOC and ^68^Ga-DOTATATE	1	10	NET	3-Tissue, Patlal	Kinetics, model comparison
^68^Ga-PSMA-11 and ^18^F-PSMA-1007	5	16–140	PC	3-Tissue, fractal	Kinetics, diagnosis
^89^Zr-DFO-MSTP2109A	1	7	PC		Kinetics
^15^C-O_2_	1	37	intraabdominal tumors	2-Tissue	Kinetics
^18^F-fluorophenylalanine	2	18–33	Brain	2-Tissue, 3-tissue, Patlak	Kinetics, model comparison, correlation to grading
^18^F-FU	1	8	Colorectal	5-Tissue	Kinetics, model comparison
^82^Rb	1	10	Brain	2-Tissue	Kinetics

*PC* prostate cancer, *HCC* hepatocellular cancer, *NSCLC* nonsmall-cell lung cancer, *MM* multiple myeloma, *GIST* gastrointestinal stromal tumor, *NET* neuroendocrine tumors, ^*11*^*C* carbon-11, ^*18*^*F* fluorine-18, ^*15*^*O* oxygen-15, ^*68*^*Ga* gallium-68, ^*15*^*C* carbon-15, ^*82*^*Rb* rubidium-82, ^*18*^*F-FDG* ^18^F-fluorodeoxyglucose, ^*18*^*F-DCFPyL* 2-(3-{1-carboxy-5-[(6-^18^F-fluoro-pyridine-3-carbonyl)-amino]-pentyl}-ureido)-pentanedioic acid, ^*18*^*F-DOPA* 6-[18F]-L-fluoro-L-3, 4-dihydroxyphenylalanine, ^*18*^*F-FAZA* 1-(5-fluoro-5-deoxy-alpha-D-arabinofuranosyl)-2-nitroimidazole, ^*18*^*F-FLT* ^18^F-fluorothymidine, ^*18*^*F-NaF* ^18^F-sodium fluoride, ^*18*^*F-EF5* 2-(2-nitro-(1)H-imidazol-1-yl)-N-(2,2,3,3,3-pentafluoropropyl)-acetamide (EF5), ^*18*^*F-FET* ^18^F-fluoro-ethyl-tyrosine, ^*18*^*F-FDHT* ^18^F-fluorodihydrotestosterone, ^*18*^*F-FMAU* (1-(2′-deoxy-2′-fluoro-beta-D-arabinofuranosyl)thymine), ^*18*^*F-FMISO* ^18^F-fluoromisonidazole, ^*18*^*F-galacto-RGD* ^18^F-galacto-arginine-glycine-aspartic acid, ^*18*^*F-HX4* (3-[18F]fluoro-2-{4-[(2-nitro-1H-imidazol-1-yl)methyl]-1H-1,2,3-triazol-1-yl} propan-1-ol), ^*68*^*Ga-DOTATOC* ^68^Ga-DOTA-Tyr3-octreotide, ^*68*^*Ga-DOTATATE* ^68^Ga-DOTA-Tyr3-octreotate, ^*68*^*Ga-PSMA-11* ^68^Ga-prostate-specific membrane antigen-HBED-CC, ^*18*^*F-PSMA-1007* ^18^F-prostate-specific membrane antigen-1007, ^*89*^*Zr-DFO-MSTP2109A* zirconium-89 desferrioxamine-MSTP2109A, ^*18*^*F-FU* ^*18*^F-fluoro-5-fluorouracil

The question raised by most physicians is when and why to use dPET imaging, given the fact that dynamic acquisition protocols are time consuming, the data processing and post-processing are complicated, and it requires dedicated software tools beyond the ones regularly provided by the manufacturers. In the following paragraphs, the main applications of dPET—additional to the conventional, static PET imaging—in several clinical settings are described.

#### Diagnosis, staging, and tumor characterization

One main clinical application of dPET is in the context of tumor diagnosis and staging. The concept behind this is the expansion of the diagnostic tools applied on the basis of a multiparametric image evaluation approach, including—besides SUV calculations—also kinetic data and parametric imaging. Several studies have highlighted the potential role of dPET imaging in initial tumor diagnosis and characterization. The vast majority of them were performed with the tracer ^18^F-FDG.

Some of the first studies applying dPET in the oncological diagnostic workup were published by Dimitrakopoulou-Strauss et al. in patients with bone tumors and soft tissue sarcomas using ^18^F-FDG. The authors demonstrated that the combination of compartmental and SUV data leads to a higher discrimination between benign and malignant lesions, as well as to a more accurate tumor grading as compared with the use of SUV alone [62, 63]. In bone tumors in particular, this multiparametric analysis was superior for the classification between grade I and grade III tumors with a positive predictive value > 80%. The mean SUV, *V*_*B*_, *K*_1_, and *k*_3_ were higher in malignant tumors compared with benign bone lesions. Overall, the combination of SUV, FD, *V*_*B*,_ *K*_1_, *k*_2_, *k*_3_, and *k*_4_ led to an accuracy of 87.7% in bone lesions as compared with 74.7% for SUV alone [62]. Regarding soft tissue tumors, *V*_*B*_ and mean SUV were higher in sarcomas as compared with benign lesions. On the basis of six parameters of the ^18^F-FDG kinetics (SUV, *V*_*B*,_ *K*_1_, *k*_3_, influx, FD), a better classification was achieved for soft tissue tumors with respect to grading as well as for differentiation between benign and malignant lesions. Interestingly, inflammatory lesions were misclassified, which was attributed to the similar ^18^F-FDG kinetics between aggressive tumors and acute inflammations [63]. In another study of the same group, it was shown that patients with giant cell tumors demonstrated significantly enhanced *V*_*B*_ as well as high *K*_1_ and FD values as compared with other tumors such as soft-tissue sarcomas, which is of interest considering the classification of giant cell tumors as benign. This result was mainly attributable to an enhanced vascular fraction and increased ^18^F-FDG transport of these tumors as was supported by gene chip data analysis, which revealed a close association between the kinetic ^18^F-FDG data and the expression of genes related to angiogenesis [52]. The additional valuable—both vascular and metabolic—information provided by dPET, not obtainable from conventional PET imaging was also highlighted in a smaller study of 11 patients with high grade (III or IV) soft tissue sarcomas. However, no significant correlations were found between the PET values and clinical factors such as tumor size, grade, and clinical status [64].

Another tumor group that has been studied by means of dPET is desmoids. Desmoids are slow-growing, locally aggressive tumors without a metastatic potential arising from the connective tissue. In patients with desmoids, it has been demonstrated that parametric imaging is helpful for the differentiation between the perfusion- and the phosphorylation-driven part ^18^F-FDG uptake. Most desmoids demonstrate a rather low to moderate ^18^F-FDG uptake, a low phosphorylation rate but a higher perfusion rate [65]. This information may have an impact on the better understanding of the tumor biology and the underlying mechanisms of tracer uptake. In addition, parametric imaging may help to acquire images with better contrast than conventional ^18^F-FDG PET images and thus improve diagnosis.

dPET has also been useful in the differentiation of primary colorectal tumors from normal colon tissue as shown in a study of 22 patients with colorectal tumors prior surgery. In particular, FD demonstrated the highest accuracy in correctly predicting both tumors and reference tissues, with a correct classification rate of 89%, which was approximately 11% higher than that reached by SUV. Moreover, the combination of all kinetic ^18^F-FDG parameters for the classification of tumors or normal colon tissue revealed an overall accuracy of 97.3% [66].

Recently, the correlation between several clinicopathological features of breast cancer and kinetic parameters measured by ^18^F-FDG dPET/CT examinations was assessed. Three-compartment kinetic modeling was applied and the parameters *K*_1_, *k*_2_, *k*_3_, *K*_*i*_ (tracer flux constant), and MRFDG (^18^F-FDG metabolic rate) were calculated. The authors confirmed a significant relationship between ^18^F-FDG kinetic parameters measured by dPET and the routinely assessed clinicopathological factors of breast cancer. In particular, high-grade, hormone-receptor negative tumors with high proliferation rate were characterized by higher cellular ^18^F-FDG uptake and phosphorylation rate [67].

In another study, Mullani et al. compared the blood flow estimated from early ^18^F-FDG images and found a linear correlation with perfusion measured by ^15^O-H_2_O (*r* = 0.86). The results suggest that a dynamic ^18^F-FDG acquisition provides additional information to tumor perfusion [68]. Comparable results have been reported by Cochet et al. in patients with breast tumors using a short early dynamic ^18^F-FDG PET acquisition (0–2 min p.i.) and late static images (90 min p.i.). The authors concluded that the perfusion-dependent part of ^18^F-FDG was significantly associated to tumor angiogenesis as evaluated by immunohistochemistry [69].

In the field of prostate cancer (PC), our group has recently studied with dynamic and static ^68^Ga-PSMA-11 PET/CT a group of 16 patients with PC biochemical relapse attributed to local recurrence. Data analysis was performed by means of two-tissue compartment as well as parametric Patlak imaging. 12/16 patients were PSMA-positive in the static scans. Early dPET as well as parametric Patlak images detected an additional PC lesion not seen in static PET/CT due to its masking form urinary bladder activity. Based on these findings, it was assumed that early PET acquisitions and parametric Patlak images may have a potential for the detection of PC local recurrence [70].

#### Therapy monitoring

PET/CT with ^18^F-FDG is an appropriate tool for therapy monitoring in a variety of tumors as well as in different therapeutic protocols including chemotherapy, radiotherapy and, most recently, immunotherapy [60, 71]. In general, response assessment is based on visual evaluation of PET images and SUV calculations as well as on the application of known response evaluation criteria for PET, like the European Organization for Research and Treatment of Cancer for PET (EORTC) and the PET response criteria in solid tumors (PERCIST) [72, 73].

Response assessment based on kinetic data has been used for research purposes in limited numbers of patients. Our group performed dPET studies mainly with the radiotracer ^18^F-FDG in patients with different tumors under several chemotherapeutic protocols. The most common observation of these studies was the overall increasing TACs prior to chemotherapy, which became decreasing or showed a plateau after treatment in responders [74, 75]. In particular, multiparametric analysis was applied in patients with metastatic colorectal cancer prior and within the course of FOLFOX chemotherapy; a combination of kinetic parameters derived from the baseline and a late follow-up ^18^F-FDG PET study (after 4 cycles) was better than the use of SUV alone for the classification of patients into a short or a long survival time (correct classification rate 78% vs. 69%) [74]. The reason for this superiority of kinetic analysis vs. conventional SUV estimations may be that kinetic data can provide an early assessment of small metabolic changes, which cannot be detected by SUV. Moreover, ^18^F-FDG dPET has been shown helpful for the therapy assessment of patients with high-risk soft tissue sarcomas receiving neoadjuvant chemotherapy, as well as of those with metastatic soft tissue sarcomas being treated with high-dose chemotherapy and peripheral blood stem cell transplantation [75–77]. Particularly in chemotherapy, the combination of SUV and influx (*K*_*i*_) in the neoaduvant setting or SUV and *K*_1_ in the adjuvant setting resulted in higher accuracy of response assessment than SUV alone (83% vs. 67%, and 90% vs. 85%, respectively) [75, 76]. In patients treated in a neoadjuvant setting with the tyrosine kinase inhibitor pazobanib, a significant decrease of the parameter *K*_1_ was demonstrated before surgery, despite the lack of a statistically significant change in SUV. This decrease in *K*_1_ was considered a potential marker in response to pazopanib due to the anti-angiogenic effect of the therapeutic agent [77]. This finding is in accordance to dPET ^18^F-FLT studies in patients with advanced solid tumors studied prior and after therapy with axitinib, a VEGFR-TK1 inhibitor. The assessment of tracer kinetics, based on 3-tissue compartment modeling, revealed a significant decrease in *V*_*B*_, *K*_1_, and *K*_*i*_ as early as 2 weeks after therapy, as a sign of an anti-angiogenic and anti-proliferative effect [78].

Mankoff et al. performed dPET studies with ^15^O-H_2_O and ^18^F-FDG in patients with locally advanced breast cancer prior and after neoadjuvant chemotherapy. They used a 2-tissue compartment for the evaluation of the perfusion studies and the metabolic rate based on Patlak graphical analysis, and reported a statistically significant trend for patients with a high metabolic rate to show a poorer response to chemotherapy. Furthermore, it was shown that a low ratio of the ^18^F-FDG metabolic rate to blood flow was predictive of disease-free survival [79]. In another dPET study in breast cancer prior and after neoadjuvant chemotherapy, Humbert et al. assessed the perfusion-related part of ^18^F-FDG using a first-pass model proposed by Mullani et al. after applying a short dynamic scan of 2 min starting with the tracer injection and a late scan 90 min p.i. [68]. Their data demonstrated a drastic reduction of the perfusion-related part of ^18^F-FDG only in HER-2 positive subtypes supporting the anti-angiogenic effect of trastuzumab. However, changes in SUVmax outperformed changes in perfusion effects for predicting pathological complete response in all tumor types [80].

Bahce et al. performed dPET studies with ^11^C-erlotinib and ^15^O-H_2_O and applied compartmental analysis in 13 patients with advanced, epidermal growth factor receptor (EGFR)-mutated nonsmall-cell lung cancer [81]. Erlotinib is a tyrosine kinase inhibitor used for treatment of EGFR-mutated tumors. A subgroup of patients was scanned twice, prior, and 1–2 weeks after beginning of erlotinib. Although no significant change in the kinetic parameters of ^15^O-H_2_O was demonstrated, the distribution volume (*V*_*B*_) of ^11^C-erlotinib decreased as response to treatment. The authors suggested that this effect may be due to the occupancy of EGF receptors by the nonlabeled erlotinib, which was given together with the tracer.

In a study by Wardak et al., dynamic longitudinal studies with either ^18^F-FLT or ^18^F-fluoro-L-DOPA were performed in 21 patients with recurrent malignant glioma prior, after 2, and 6 weeks after onset of treatment with bevacizumab (an angiogenesis inhibitor) and irinotecan (a chemotherapeutic agent). They showed that ^18^F-FLT kinetic parameters early after onset of treatment were more predictive for overall survival than SUV. On the other hand, ^18^F-fluoro-L-DOPA information was inferior to ^18^F-FLT [82].

In multiple myeloma (MM), a prospective study in 19 patients undergoing ^18^F-FDG PET/CT before and after the first cycle of chemotherapy showed that changes in SUV and kinetics of the radiotracer could predict progression-free survival and identify patients who mostly benefited from therapy [83]. Furthermore, in a group of 34 MM subjects undergoing high-dose chemotherapy and autologous stem-cell transplantation PET/CT studies with ^18^F-FDG and the skeletal imaging tracer ^18^F-NaF were performed before and after treatment. It was observed that SUV as well as kinetic parameters of ^18^F-FDG and ^18^F-NaF significantly decreased in all patients, who at the same time showed at least partial remission of the disease according to the clinical gold standard [84].

Another interesting field in terms of oncological therapy assessment is immunotherapy. The recent introduction and increasing application of immunotherapeutic agents in clinical practice has resulted in unprecedented improvements in patients’ survival. Due to their unique mechanism of action, these novel agents have been associated with atypical response patterns by means of standard criteria. Thus, the application of conventional response criteria may misinterpret the effectiveness of immunotherapy. In an attempt to address this issue, dPET has also been employed. Nevertheless, the initial results were not satisfying. In particular, in patients suffering from stage IV metastatic melanoma being treated with ipilimumab immunotherapy, no superiority of dynamic ^18^F-FDG PET/CT as compared with static images could be demonstrated [85]. In this cohort of patients, the best criterion for immunotherapy response assessment was the number of new lesions detected on serial PET/CT imaging [86, 87]. This fact may be melanoma-specific and related to the fact metastatic melanoma tends to metastasize rapidly in case of disease progression. Another recently proposed PET-based approach for prediction of eventual response in advanced melanoma patients under immunotherapy is the combination of anatomic (CT) and functional (PET) imaging parameters including SUV changes [88].

Overall, a multiparametric PET/CT evaluation based on a combination of SUV and kinetic data seems to be promising and superior to an assessment based merely on SUV calculations. Moreover, it is expected that the implementation of artificial intelligence in medical practice will facilitate and improve multiparametric approaches.

#### Pharmacokinetic studies for tracer characterization

Another main application of dPET is the pharmacokinetic characterization of new tracers. This can only be done by the use of dynamic scanning over a certain time depending on radiotracer studied. Most such studies have been performed either in preclinical models or in the field of neurosciences, for example, pharmacokinetic studies of dopamine, nicotinic acetylcholine or serotonin receptors as well as brain perfusion studies [89–92]. In the field of clinical oncology, besides ^18^F-FDG, more recently radiolabeled peptides have also been successfully studied with dPET/CT, such as somatostatin analogues (e.g., ^68^Ga-DOTATOC and ^68^Ga-DOTATATE) or PSMA radioligands (e.g., ^68^Ga-PSMA-11 and ^18^F-PSMA-1007). In the case of receptor-binding tracers, a two-tissue compartment model can be used as a simplification for the calculation of the rate constants and *V*_*B*_. The interpretation of the rate constants is however different than that of ^18^F-FDG, with *K*_1_ reflecting the receptor binding, *k*_2_ the displacement from the receptor, *k*_3_ the cellular internalization, and *k*_4_ its externalization.

Neuroendocrine tumors (NETs) of the gastrointestinal tract as well as meningiomas are ^68^Ga-DOTATOC avid. It has been demonstrated that untreated patients with these tumors show a continuous increase of ^68^Ga-DOTATOC [17, 93]. Koukouraki et al. reported that *K*_1_ had the greatest impact on the global ^68^Ga-DOTATOC SUV in NET followed by *V*_*B*_ and *k*_3_. Overall, pharmacokinetic analysis helped to separate blood background activity from receptor binding, which may have an impact for radionuclide therapy planning [17]. Ilan et al. compared the *K*_*i*_ values based on kinetic data of a 3-tissue compartment model and the ones from parametric Patlak images for ^68^Ga-DOTATOC and ^68^Ga-DOTATATE in patients with metastatic NET and found a very high agreement [94]. In another study higher *K*_1_/*k*_2_ and *k*_3_/*k*_4_ values for ^68^Ga-DOTATOC in meningiomas as compared with reference tissue, as well as high *V*_*B*_ values were reported, highlighting the more detailed analysis of the tumor biologic properties offered by pharmacokinetic modeling [93].

Sachpekidis et al. performed pharmacokinetic studies in PC patients with both ^68^Ga-PSMA-11 and ^18^F-PSMA-1007 [16, 95]. They reported on a continuous tracer increase up to 60 min as well as significantly higher kinetic values of ^68^Ga-PSMA-11 and ^18^F-PSMA-1007 in prostatic recurrence and metastatic lesions as compared with reference tissues. Comparable results have been reported by Schmuck et al. with ^68^Ga-PSMA-11 and a short acquisition protocol (0–10-min dynamic acquisition and two late static images at 60 and 180 min p.i.) in 20 patients with primary PC. The authors concluded that early and delayed ^68^Ga-PSMA-11 images best discriminate PC within the prostatic gland [96]. Overall, the results are indicative for a high receptor binding and internalization of ^68^Ga-PSMA-11 and ^18^F-PSMA-1007 in prostate tumors and metastases, which may have potential applications in the field of PSMA radioligand therapy.

Pharmacokinetic results have been reported with the ^68^Ga-bombesin analog BZH_3_, which is a pan-bombesin analog that binds to at least three receptor subtypes, the neuromedin B (or BB_1_), the gastrin-releasing peptide or GRP (or BB_2_), and the bombesin receptor subtype 3 (BB_3_). dPET studies with ^68^Ga-BZH_3_ in patients with gastrointestinal stromal tumors (GISTs) demonstrated an enhanced accumulation in 41% of the patients and overall lower SUV and kinetic values for ^68^Ga-BZH_3_ as compared with ^18^F-FDG. The authors of that study concluded, that ^68^Ga-bombesin may be useful in a subgroup of GIST patients with low proliferation rate and therefore negative in ^18^F-FDG PET [97]. In addition, in patients with recurrent gliomas ^68^Ga-BZH_3_ seems to be helpful for the differentiation between low- and high-grade gliomas based on a combination of kinetic ^18^F-FDG and ^68^Ga-BZH_3_ data. Overall, the ^68^Ga-BZH_3_ accumulation was lower as compared with ^18^F-FDG [98].

A plethora of other pharmacokinetic studies with different radiotracers by means of dPET has been performed in patients with several malignancies. For example, ^18^F-fluroethyltyrosine (FET) and ^18^F-FLT have been used for the diagnosis of patients with gliomas [99, 100], while ^18^F-fluromisonidazole (MISO) has been successfully tested in different tumor entities like lung tumors and head and neck tumors for the determination of the hypoxic parts of the tumors [101–103]. Further, kinetic modeling has been used for the characterization of new tracers like ^18^F-fluciclovine and ^18^F-HX4 [104, 105].

### Future perspectives

dPET scanning is helpful for the diagnosis and therapy monitoring of oncological patients, but it is time-consuming and requires more complex evaluation techniques, which may hamper its routine use. Issues that are still open and need to be addressed include the following:

- The definition of shorter acquisition protocols including, e.g., a short dynamic acquisition immediately after tracer injection for the calculation of the input function and a short late dynamic acquisition 50–60 min p.i. We proposed a short dynamic acquisition 0–16 min and a late acquisition 60 min p.i. for ^18^F-FDG and could demonstrate a high correlation between the kinetic data obtained from this short acquisition protocol as compared with a full dynamic series over 60 min [106]. Moreover as mentioned above, Karakatsanis et al. proposed a 7-frame protocol, consisting of an initial early dynamic scan (6 min) at the cardiac bed position followed by 6 whole-body dynamic scans over seven bed positions (40 min) [46].
- The improvement of the evaluation software for dynamic images including sophisticated segmentation algorithms, automatic VOI placement, automatic calculation of TACs, implementation of validated methods for 2- and 3-tissue compartment modeling including a graphical interface for the users.
- Automatic calculation of the input function, either image-derived or population-based.
- Faster acquisition protocols and a potential implementation of whole-body parametric imaging, e.g., Patlak or simplified parametric model-based analysis for the new generation PET/CT scanners with an extended FOV.
- New reconstruction algorithms for the new generation PET/CT scanners to improve counting statistics and image resolution. This issue can, however, be addressed with the introduction of clinically feasible dynamic whole-body PET imaging protocols in current generation limited axial FOV PET systems equipped with direct 4D reconstruction schemes and generalized nonlinear graphical analysis methods [8, 10, 107, 108].
- Implementation of artificial intelligence for the image analysis in order to facilitate and improve multiparametric approaches (combination of kinetic modeling and SUV measures).

The implementation of such quantitative approaches will need to be optimized, but it will open a new era for PET imaging. The adoption of such dynamic whole-body protocols, including parametric imaging, would facilitate the use of dPET initially in clinical studies for dedicated questions, and afterwards even into routine clinical protocols. Prerequisites for this wider usage of dPET include a further evidence of the added value and the gain in information offered by dPET compared with conventional static PET alone, and its acquisition in a patient- and operator-friendly manner.

#### Limitations

Dynamic PET/CT imaging for oncology including kinetic modeling and parametric imaging has been used until now primarily for research purposes and cannot be yet recommended for clinical use in its present form due to several limitations. Kinetic modeling needs further optimization to avoid overfitting, for example with the use of reference databases and SVM algorithms, as well as a robust definition of the input VOI. Furthermore, parametric images are noisy; thus, their interpretation should be done by experienced users and in comparison with conventional SUV images for reference. This limitation, however, is beginning to be addressed with the direct 4D parametric PET image reconstruction that has been introduced for single-bed and multibed dynamic PET studies, and which may allow the generation of parametric PET images of similar noise levels to those of conventional SUV images. In line with this, the industry has recently automated this technique to offer the automatic generation of direct Patlak parametric image and image-derived plasma input functions from dPET whole-body PET data. This effort represents a promising solution to facilitate the clinical translation of dPET imaging to clinical routine in oncology PET studies. Finally, another limitation is that although parametric imaging based on the Patlak approach and the simplified parametric analysis of dPET data have been thoroughly investigated in an experimental level, data are lacking on other algorithms like compartment modeling, FD, PCA, and SM. Overall, kinetic modeling and parametric imaging need further development and optimization of the algorithms used for calculation prior to their introduction in clinical practice.

## Conclusion

Multiparametric dPET based on kinetic modeling and parametric imaging of the applied radiotracers, offers a plethora of data not otherwise acquired with the conventional, static PET/CT. Its introduction in the diagnostic approach of the oncological patient is expected to provide superior information than the one derived from the visual evaluation of PET images merely supported by one semi-quantitative parameter, namely SUV. Although at present confined to research protocols, quantitative dPET and parametric imaging may gain importance and find increasing usage in the clinical routine as long as certain issues are addressed. This would be probably accomplished with the advent of the new generation PET/CT scanners and the expected improvement of the technical equipment, including an extended FOV, faster data acquisition and more sophisticated software for data evaluation.
